# Effect of Transcranial Direct Current Stimulation Combined With Patient-Controlled Intravenous Morphine Analgesia on Analgesic Use and Post-Thoracotomy Pain. A Prospective, Randomized, Double-Blind, Sham-Controlled, Proof-of-Concept Clinical Trial

**DOI:** 10.3389/fphar.2020.00125

**Published:** 2020-02-25

**Authors:** Dusica M. Stamenkovic, Katarina Mladenovic, Nemanja Rancic, Vlado Cvijanovic, Nebojsa Maric, Vojislava Neskovic, Snjezana Zeba, Menelaos Karanikolas, Tihomir V. Ilic

**Affiliations:** ^1^ Department of Anesthesiology and Intensive Care, Military Medical Academy, Belgrade, Serbia; ^2^ Medical Faculty Military Medical Academy, University of Defense, Belgrade, Serbia; ^3^ Center for Clinical Pharmacology, Military Medical Academy, Belgrade, Serbia; ^4^ Clinic for Cardiothoracic Surgery, Military Medical Academy, Belgrade, Serbia; ^5^ Department of Anesthesiology, Washington University School of Medicine, St. Louis, MO, United States; ^6^ Department of Neurology, Military Medical Academy, Belgrade, Serbia

**Keywords:** transcranial direct current stimulation, randomized double-blind study, prospective study, pain management, acute pain, analgesia

## Abstract

**Background:**

Transcranial direct current stimulation (tDCS) is used for various chronic pain conditions, but experience with tDCS for acute postoperative pain is limited. This study investigated the effect of tDCS vs. sham stimulation on postoperative morphine consumption and pain intensity after thoracotomy.

**Methods:**

This is a single-center, prospective, randomized, double-blind, sham-controlled trial in lung cancer patients undergoing thoracotomy under general anesthesia. All patients received patient-controlled (PCA) intravenous morphine and intercostal nerve blocks at the end of surgery. The intervention group (a-tDCS, n = 31) received anodal tDCS over the left primary motor cortex (C3-Fp2) for 20 min at 1.2 mA, on five consecutive days; the control group (n = 31) received sham stimulation. Morphine consumption, number of analgesia demands, and pain intensity at rest, with movement and with cough were recorded at the following intervals: immediately before (T1), immediately after intervention (T2), then every hour for 4 h (Т3–Т6), then every 6 h (Т7–Т31) for 5 days. We recorded outcomes on postoperative days 1 and 5 and conducted a phone interview inquiring about chronic pain 1 year later (NCT03005548).

**Results:**

A total of 62 patients enrolled, but tDCS was prematurely stopped in six patients. Fifty-five patients (27 a-tDCS, 28 sham) had three or more tDCS applications and were included in the analysis. Cumulative morphine dose in the first 120 h after surgery was significantly lower in the tDCS [77.00 (54.00–123.00) mg] compared to sham group [112.00 (79.97–173.35) mg, p = 0.043, Cohen’s d = 0.42]. On postoperative day 5, maximum visual analog scale (VAS) pain score with cough was significantly lower in the tDCS group [29.00 (20.00–39.00) vs. 44.50 (30.00–61.75) mm, p = 0.018], and pain interference with cough was 80% lower [10.00 (0.00–30.00) vs. 50.00 (0.00–70.00), p = 0.013]. One year after surgery, there was no significant difference between groups with regard to chronic pain and analgesic use.

**Conclusion:**

In lung cancer patients undergoing thoracotomy, three to five tDCS sessions significantly reduced cumulative postoperative morphine use, maximum VAS pain scores with cough, and pain interference with cough on postoperative day 5, but there was no obvious long-term benefit from tDCS.

## Introduction

Thoracotomy is a painful incision that involves multiple muscle layers, rib resection and pain is exacerbated by ongoing continuous movement due to breathing (Gerner, 2008). In addition, published data suggest that acute post-thoracotomy pain can influence the appearance and intensity of chronic post-thoracotomy pain (Katz et al., 1996; Bayman et al., 2017; Kampe et al., 2017).

Thoracic epidural analgesia is considered as gold standard for pain after thoracotomy, whereas systemic analgesia is used in patients not eligible for epidural analgesia (Gottschalk et al., 2006; Kampe et al., 2013; Kampe et al., 2014; Maxwell and Nicoara, 2014). Because multimodal analgesia regimens include pharmacological agents with potential for significant adverse events, there is an opportunity in post-thoracotomy pain management for development of new, improved techniques with fewer adverse events.

Transcranial direct current stimulation (tDCS) is a noninvasive cortical stimulation technique with neuromodulatory effects, altering cortical excitability through subthreshold modulation of neuronal resting membrane potentials by constant weak electrical current (Nitsche et al., 2003a; Nitsche et al., 2008; Stagg and Nitsche, 2011).

The proposed mechanism of pain alleviation by tDCS is based on modulation of cortical excitability in locations that can be considered as entry points for the wider areas of neuronal networks, the so-called “pain matrix” (Ayache et al., 2016). However, in addition to this mechanism, recent evidence indicates an interaction of tDCS with a number of neurotransmitter systems (serotonin, dopamine, GABA, acetylcholine) (Knotkova et al., 2013), as well as changes in serum brain-derived neurotrophic factor (BDNF) levels, which also take part in the processing of painful stimuli (Stefani et al., 2012). Furthermore, in addition to local effects in the area of stimulation, significant changes in remote connected areas related to processing of motor, cognitive, or pain information have also been demonstrated (Stagg et al., 2013).

Published data suggest that tDCS, when used as part of multimodal postoperative analgesia can result in reduced postoperative opioid use and reduced pain in patients undergoing endoscopic retrograde cholangiopancreatography (ERCP) (Borckardt et al., 2011), total knee arthroplasty (Borckardt et al., 2013; Borckardt et al., 2017; Khedr et al., 2017b), and hallux valgus surgery (Ribeiro et al., 2017), while results for patients who underwent lumbar spine surgery are equivocal (Dubois et al., 2013; Glaser et al., 2016).

Advantages of tDCS use for postoperative analgesia include simplicity of use, patient comfort, absence of a magnetic field, and low cost, and therefore tDCS is a promising option as non-pharmacological addition to a multimodal postoperative analgesia regimen (Borckardt et al., 2009; Bikson et al., 2016; Antal et al., 2017). Furthermore, tDCS with standard parameters seems to be safe, and the combination of tDCS with pain medications has not been associated with significant safety issues (Antal et al., 2017).

The primary objective of this single-center, prospective, randomized, double-blind clinical trial is to evaluate the effect of anodal tDCS combined with patient-controlled analgesia (PCA) morphine, on intravenous (IV) morphine consumption for analgesia after thoracotomy. The intervention group received treatment with anodal tDCS, whereas the control group received sham stimulation. We hypothesized that tDCS will result in reduced postoperative morphine use (primary outcome) and lower postoperative pain intensity at rest, with movement and with cough (secondary outcome) in patients receiving IV morphine PCA for analgesia after thoracotomy.

## Methods

### Study Design and Patient Selection

This prospective, randomized, double-blinded sham/controlled study was carried out in the Department of Cardiothoracic Surgery and the Department of Anesthesia and Intensive Care at the Military Medical Academy in Belgrade (Serbia) in the period from June 15, 2016 to March 27, 2018. The study was approved by the Institution Ethics Committee and was registered in Clinical Trials: https://clinicaltrias.gov (registration number NCT03005548). All eligible patients received detailed information about the study protocol and goals and gave written consent before enrolling in the study.

**Figure 1:**
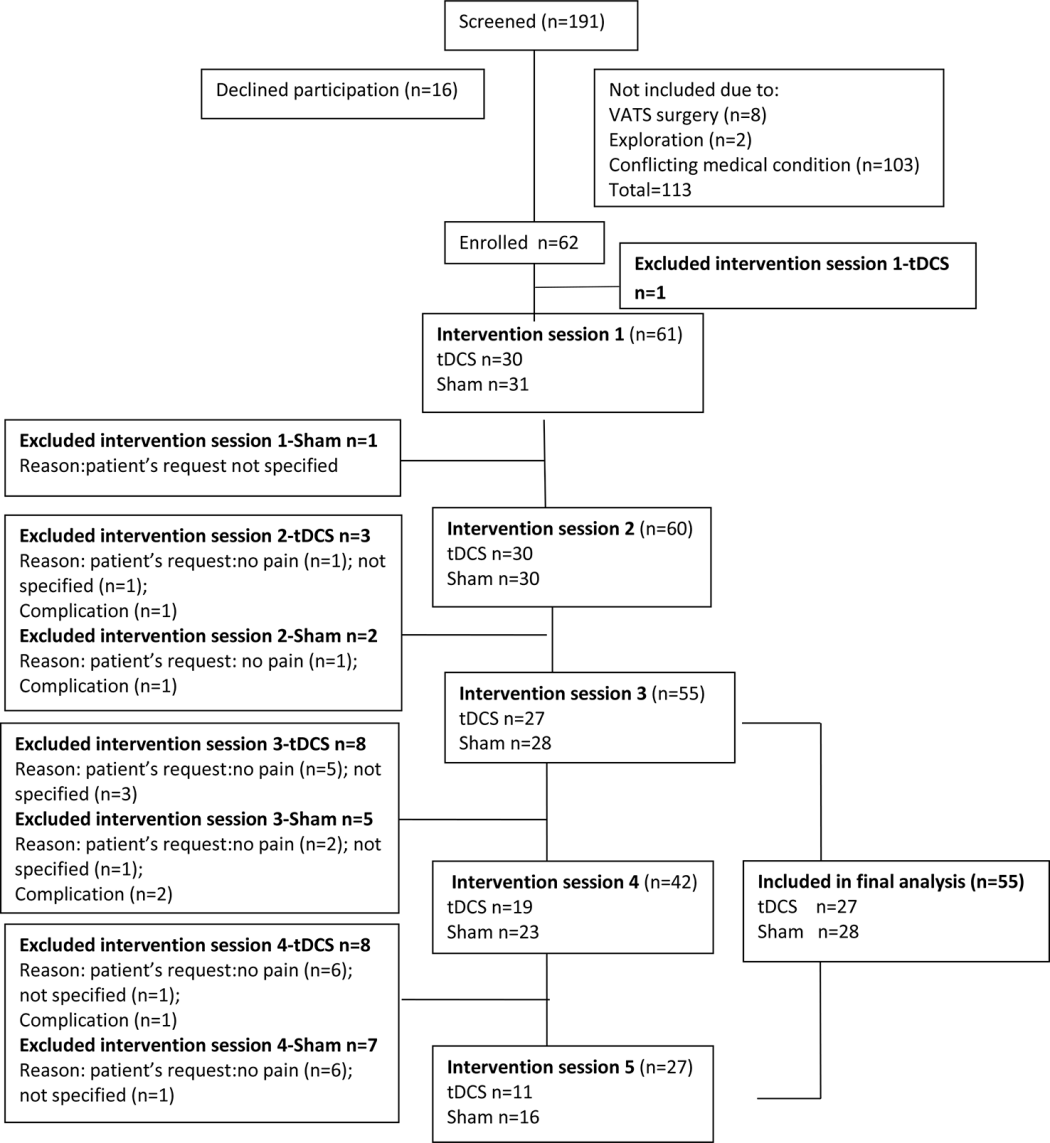
Study flowchart. VATS, video-assisted thoracoscopic surgery; tDCS, transcranial direct current stimulation.

Inclusion criteria were patient willingness to participate, ability to understand the protocol and provide written informed consent, age 18–80, scheduled thoracotomy for confirmed primary malignant lung disease, and planned tracheal extubation in the operating room immediately after surgery. Exclusion criteria were pregnancy, treatment for neurological or psychiatric diseases, any chronic pain condition, history of alcohol or drug abuse, chemotherapy, history of previous thoracic or cardiac surgery, allergy to medications used in the study, presence of pacemaker, automatic implantable cardioverter/defibrillator or any other implanted device in the head, spinal cord, or peripheral nerves, and confirmed brain lesion, including tumor or metastasis (Woods et al., 2016). The flowchart of the study is presented in **Figure 1**.

Patients in both groups received intraoperative IV morphine followed by postoperative IV morphine PCA (bolus 1 mg, lockout time 10 min) using the CADDLegacy PCA Pump (Deltec, Inc.). Patients assigned to the active treatment group (a-tDCS, n = 31), received tDCS (20 min of 1.2 mA anodal tDCS over the left primary motor cortex for 5 days), whereas patients assigned to the sham control group (sham-tDCS, n = 31) received sham tDCS stimulations over the left primary motor cortex for 5 days.

### Sample Size Calculation

Sample size calculation was conducted using the freely available from the University of Dusseldorf, Germany, G*Power statistical program v. 3.1.9.2 (Faul et al., 2007) and was based on the following assumptions: two tailed t-test, beta error = 0.2 (power of 80%), alpha = 0.05, mean morphine consumption 39 mg in one group vs. 62 mg in the other group with SD = 30, based on previously published data (Borckardt et al., 2008). Based on these assumptions, the required sample size would be 28 patients per group. Therefore, because we assumed 10% overall attrition rate, we decided to increase the sample size by 10% to 31 patients per group.

Participating patients were instructed to continue the study for 5 days. Criteria for early discontinuation of the study included the absence of any pain, patient request (for any reason), adverse analgesic medication or tDCS effects and any complications requiring prolonged postoperative intubation, mechanical ventilation, or additional surgical interventions.

### Randomization and Blinding

In order to preserve blinding, only one independent physician (TVI) was in charge of the randomization procedure and adjusted tDCS in either anodal or placebo mode but had no contact with the patient and was not involved in any stage of collecting or processing data. All other investigators were blinded to group assignment. The tDCS stimulation session was performed by an investigator who had no knowledge of other aspects of patient data. After the first tDCS session, all patients were asked if they thought they were receiving active or sham stimulation. Analysis of their response did not show any significant findings, therefore we concluded that the blinding procedure was probably successful.

Group allocation was masked as group 1 or 2 for statistical analysis. Group allocation to the active tDCS (intervention) group or the sham (control) group was revealed after final data analysis.

A computer-generated permuted block randomization method (1:1) was used to allocate patients to the active or sham tDCS groups based on order of inclusion in the study, in order to ensure concealment.

### Intervention

tDCS was transmitted through two circular Ag/AgCl electrodes (1 cm radius) with conductive gel fixed by the neoprene head cap and was delivered by a battery-driven, wireless Starstim tDCS neurostimulator (Neuroelectrics, Barcelona, Spain). The anode was placed over the left primary motor cortex, Brodmann area 4 (C3 position of the International 10-20 electroencephalogram electrode system), and the return electrode was placed over the contralateral supraorbital region (Fp2). The anodal tDCS group received stimulation for 20 min at 1.2 mA (current density 0.38 mA/cm^2^; charge density 0.127 mAh/cm^2^) per session. In sham stimulations, current was applied over the same electrode montage for 60 s with a ramp time of 10 s and then gradually turned off at the start of the 20-min period. At the end of the period, the current was ramped up slowly and then turned off more quickly (Nitsche et al., 2003b).

### Anesthesia, Surgery, and Perioperative and Postoperative Pain Management Protocol

Patients were screened for possible inclusion in the study the day before surgery. Then, an investigator approached each eligible patient and provided information about the study, perioperative course, the expected level of acute postoperative pain, and incidence of chronic pain after thoracotomy. After signing a written consent, patients were instructed on the use of the visual analog (VAS) for assessment of pain and on the use of the PCA pump and received additional information about the tDCS procedure. In addition, on the morning of surgery patients were once again checked to ensure that they understand the pain assessment score and had their VAS for anxiety and depression assessed. After arrival in the operating room, standard monitoring was placed, IV access was obtained, and midazolam 2 mg was given as premedication. General anesthesia was induced using target-controlled infusion (TCI) of remifentanil (1–5 ng ml^−1^) and propofol (5 µg ml^−1^), while cis-atracurium (0.2 mg kg^−1^) was given for muscle relaxation. After induction, placement of double lumen endotracheal tube and confirmation of appropriate tube position with bronchoscopy, anesthesia was maintained with TCI of remifentanil (1–5 ng ml^−1^) and propofol (5 µg ml^−1^), while cis-atracurium (0.03 mg kg^−1^) was given as needed to maintain neuromuscular blockade. Entropy levels (Datex-Ohmeda S/5™ Anesthesia Monitor, GE Healthcare Finland Oy, Helsinki, Finland) were maintained in the 40–60 range throughout the procedure and FiO_2_ was set at 80% in order to keep SaO_2_ > 92%.

Thoracotomy was performed using a conventional anterolateral thoracotomy approach (Ferguson, 2007). The skin incision was 15 to 20 cm long, parallel to the ribs at the lateral part of the fifth intercostal space. The fifth intercostal space was crossed by subperiosteal rib resection along the superior border of the sixth rib. Rib spreader was used for two ribs retraction, with the superior part of the spreader moving apart the full content of the intercostal space (including the fifth intercostal nerve) against the inferior border of the fifth rib. The procedure was lobectomy or pneumonectomy depending on intraoperative findings.

During surgery, after resection of the lobe or lung was completed, we applied positive pressure to the bronchial stump and lobe in order to confirm the absence of significant postoperative air leak (“air leak test”) (Fell SC and Feins, 2018). After the “air leak test” morphine 2 mg was given as a bolus, morphine infusion was started at 0.01–0.05 mg/kg/h and paracetamol 1000 mg was infused over 30 min. At the beginning of chest closure, the surgeon performed ipsilateral intercostal blocks under direct vision at T4 to T7 levels using levobupivacaine 0.5%, 2 ml per level. At the end of surgery, intercostal drains were placed, and the intercostal space was closed by peri-costal sutures. After the end of surgery patients were extubated and transferred to the Post-Anesthesia Care Unit.

Postoperatively, as soon as VAS at rest was below 30 mm, tDCS was started and each patient was switched to morphine IV-PCA (bolus 1 mg, lockout time 10 min) with a dedicated PCA pump (CADDLegacy PCA Pump, Deltec, Inc., Ashford, Kent, UK). For patient convenience PCA morphine bolus was increased from 1 to 2 mg based on the individual increased number of attempted requests recorded with pump. Patients received IV morphine PCA for 5 days, but PCA was continued beyond postoperative day 5 if needed, but could also be discontinued earlier if no longer needed or at patient request. At the ward, all patients were monitored by pulse oximetry. If analgesia was inadequate (VAS score in rest ≥30 mm), the IV line was checked, additional morphine 1–2 mg was given as bolus, and analgesia was supplemented with IV diclofenac 75 mg twice a day and a single 1000 mg dose of IV paracetamol. After discontinuation of the IV PCA, patients received IV paracetamol 1000 mg every 6 h. After hospital discharge, patients received a prescription for oral nonsteroidal analgesics or paracetamol but not for any opioids. For the purposes of this study, data were collected during the first five postoperative days.

### Outcomes and Assessments

The primary outcome of this study was the amount of morphine used for analgesia after thoracotomy in a group of patients receiving tDCS in comparison with the amount of morphine used for analgesia in patients receiving sham stimulation for up to 5 days.

Secondary outcomes were pain scores measured using VAS (from 0 no pain to 100 mm the worst possible pain) at rest, during movement, and during cough in patients receiving tDCS and IV morphine PCA, compared to patients receiving sham stimulation and IV morphine PCA.

**Figure 2:**
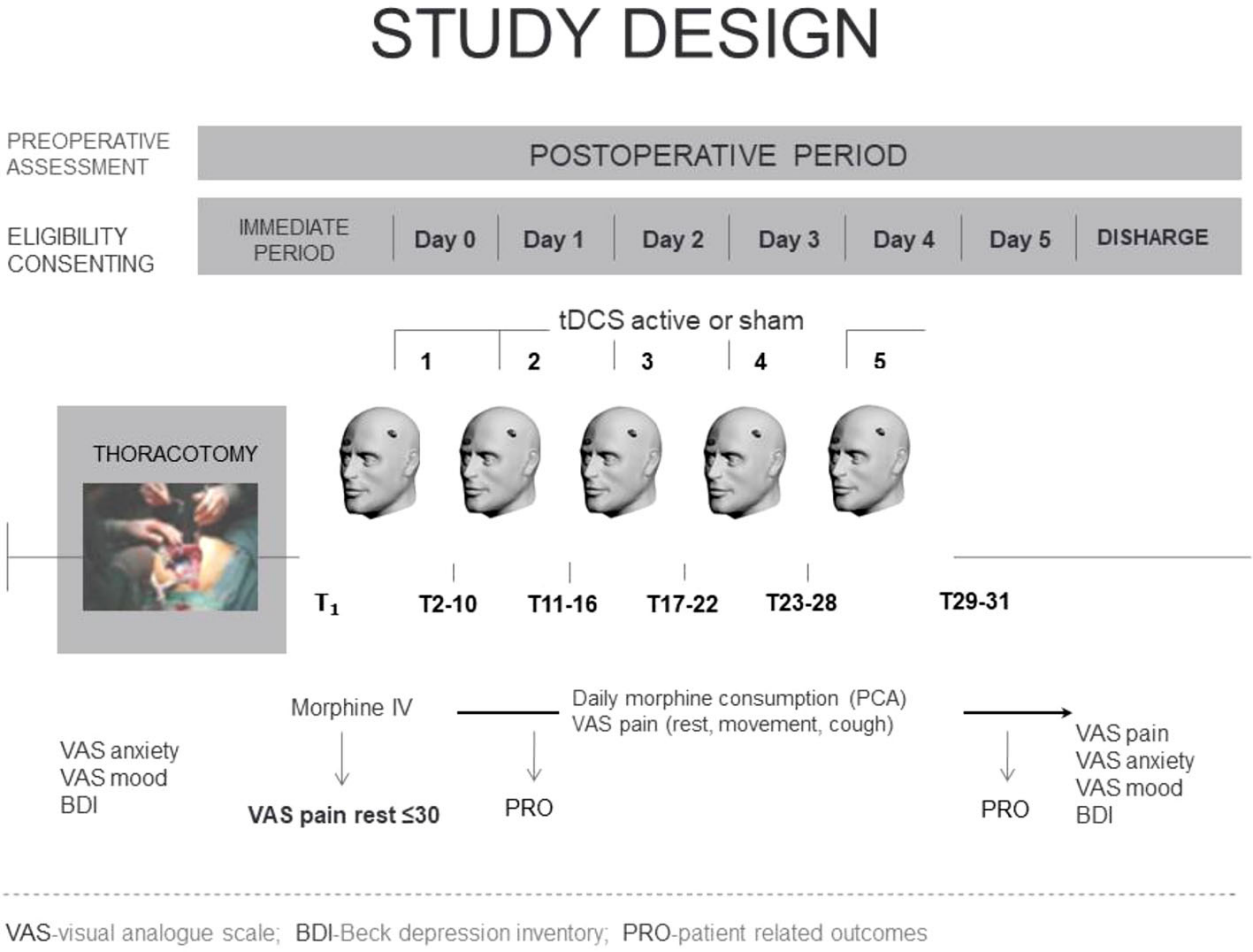
Study design.

After surgery, the first tDCS session was applied when the VAS pain score at rest fell below 30 mm. Morphine consumption, the number of analgesia demands, and pain intensity at rest, with movement and with cough were recorded at predetermined time intervals as follows: immediately before the intervention (T1), immediately after the intervention (T2), then regularly every 1 h for 4 h (Т3–Т6), and then every 6 h (Т7–Т31) for 5 days (**Figure 2**).

A researcher blinded to group assignments evaluated all patients in the morning on postoperative day 1 and postoperative day 5 using the patient-related outcomes (PRO) survey (Rothaug et al., 2013). The PRO survey consists of questions including the intensity of “the worst possible pain” (graded from 0 to 100); the percentage of time with severe pain (graded from 0 to 100%); pain interference with activity, cough, sleep, and mood (graded from 0 to 100); and patient satisfaction (0 to 100). The PRO survey was completed by the patients themselves; the researcher was only responsible for ensuring that all questions were answered. Baseline pain, anxiety, and depression were assessed on the morning of surgery and on the day of discharge from the hospital. Maximum VAS pain scores (i.e., the highest pain score number reported by each patient in each time frame) at rest, with movement and with cough were recorded. Demographic data and comorbidities were also recorded.

Baseline anxiety level was assessed with VAS for anxiety (Facco et al., 2013), whereas depressive symptoms were assessed with the Beck Depression Inventory (Beck et al., 1996). In addition, we recorded postoperative complications (surgical complications, allergy, pruritus, nausea, vomiting, hypotension, respiratory depression, delirium, weakness) and tDCS complications (headache, tiredness, nausea, tingling, itching sensations under the electrodes) (Stagg and Nitsche, 2011; Woods et al., 2016).

Management of the chest tube was at the discretion of the surgeon. Based on the publication by Bayman et al. (2017) a drain was recorded as “present” if it was present at 7:00 am during morning rounds. Respiratory complications were defined based on a study by Canet et al. (2010) and extrapulmonary complications were recorded based on the definitions of the PROVIHLO trial (Hemmes et al., 2014).

During hospitalization, after discontinuation of the intervention we recorded opioid analgesia requests besides regular paracetamol therapy and the amount of morphine used. If patients agreed to be contacted, a phone interview was conducted 1 year after surgery. The interview contained questions about presence of chronic pain after surgery; if pain was present, additional questions included time when pain appeared, medications used for pain treatment, and the influence of pain on daily activity. Because of concern about possible adverse effects of tDCS, we prepared a structured questionnaire in accordance with questionnaire surveys for tDCS adverse effects (Brunoni et al., 2011), and administered the questionnaire to all patients after each individual tDCS or sham session.

### Statistical Analysis

In total, 55 patients who received three or more tDCS stimulation sessions were included in data analysis (**Figure 1**). Data were analyzed “per protocol” based on tDCS cumulative effect. Categorical variables [sex, American Society of Anesthesiologists Physical Status (ASA) score, smoking, comorbidities, surgery type, postoperative complications, reasons for tDCS termination, number of tDCS sessions, number of patients using nonopioid drugs and pharmacological treatment for chronic pain, and number of patients with chronic pain] were presented as frequency and were analyzed using the chi-square test. All continuous variables [age, body mass index (BMI), surgery duration, use of remifentanil, propofol and pre tDCS morphine loading, cumulative morphine dose, VAS, hospitalization duration, drain, anxiety level, mood, and Beck depression scale score] are presented as mean ( ± SD) for normally distributed data or median [interquartile range (IQR): 25–75 percentile] for non-normally distributed data. The Shapiro–Wilk test was used to test the normality of data distribution. For intergroup (active vs. sham) comparisons, the independent t-test was used for parametric variables (age, BMI, surgery duration, use of remifentanil, propofol, and pre-tDCS morphine loading), and the Mann–Whitney U test for non-parametric variables (cumulative morphine dose, VAS, hospitalization duration, drain, anxiety level, mood, and Beck depression scale score). The relationship between variables was evaluated using the Pearson’s coefficient correlation. Cohen’s d for the mean difference also was performed for a continuous variable of interest (cumulative morphine dose). Statistical analysis was conducted using IBM SPSS Statistics, version 19.0 (SPSS, Chicago, IL, USA) and statistical significance was defined as p < 0.05 for all comparisons. Missing values were less than 5%.

The period between two consecutive stimulations was used as a time frame for data analysis, starting with the time frame between the first and the second session, the second and the third, and so on, and therefore there were five time frames: time after the first session, after the 2^nd^ tDCS, after the 3^rd^ tDCS, after the 4^th^ tDCS, and after the 5^th^ tDCS session. Morphine dose was calculated as cumulative dose before every preceding stimulation, i.e., the dose of morphine between two consecutive stimulations, including the period 24 h after the last tDCS stimulation. VAS pain scores were recorded in three different conditions: at rest, with movement, and with cough at regular time intervals between consecutive tDCS sessions. The highest VAS score among these measurements for the individual patient served as representative for that time frame. Then, we calculated and presented a median (IQR) of maximum VAS pain scores of all patients in the group for a particular time frame. The same principle was applied separately for pain in different conditions.

## Results

Overall 62 patients enrolled in the study. One patient was excluded because he required emergency re-operation. tDCS was prematurely stopped in six patients due to surgical complications (n = 2), absence of pain (n = 2), or per patient request without a specific reason (n = 2). Patients were included in the analysis only if they received three or more tDCS applications, and data were analyzed “per protocol” (27 in the tDCS and 28 in the sham group, 55 in total, as shown in **Figure 1**).

Baseline demographic data (**Table 1**), comorbidities (p = 0.525), intraoperative data (**Table 2**), and surgery type (p = 0.383) (**Table 2**), were not significantly different between groups. Maximum VAS pain scores at rest before intervention were not significantly different between the tDCS and the sham group [20.00 (10.00–29.00) vs. 29.00 (22.00–29.75), p = 0.102].

**Table 1:** Demographic information.

Variable	Active group (n = 27)	Sham group (n = 28)	p Value
**Age (years)**	61.44 (7.98)	61.89 (5.79)	0.812^a^
**BMI (kg m^-2^)**	26.01 (4.34)	25.81 (4.89)	0.873^a^
**Sex (female/male)**	11/16	5/23	0.116^b^
**ASA (2/3)**	4/23	3/25	0.959^b^
**Smoking at present**	12 (44.4%)	15 (53.6%)	0.248^b^

Data are presented as mean (SD) or number (percentage). Data analysis did not show any significant differences between the active vs. sham group.

BMI, body mass index; ASA, American Society of Anesthesiologists Physical Status.

a Independent t-test.

b Chi-square test.

**Table 2:** Intraoperative data, presented as mean (SD) or number of cases.

Variable	Active group (n = 27)	Sham group (n = 28)	p Value
**Surgery duration (min)**	144.44 (33.16)	152.36 (33.27)	0.381^a^
**Remifentanil (μg)**	646.87 (350.29)	751.04 (552.47)	0.409^a^
**Propofol (mg)**	1290.72 (411.65)	1306.18 (466.45)	0.897^a^
**Pre tDCS morphine loading (mg)**	16.28 (6.75)	17.43 (6.96)	0.537^a^
**Surgery type**			
Lobectomy	23	19	0.383^b^
Pneumonectomy	4	9	

Data analysis did not show any significant differences between the active vs. sham group. Pre-tDCS morphine loading is the amount of morphine needed to achieve visual analog scale (VAS) pain ≤30 at rest.

tDCS, transcranial direct current stimulation.

a Independent t-test.

b Chi-square test.

**Figure 3:**
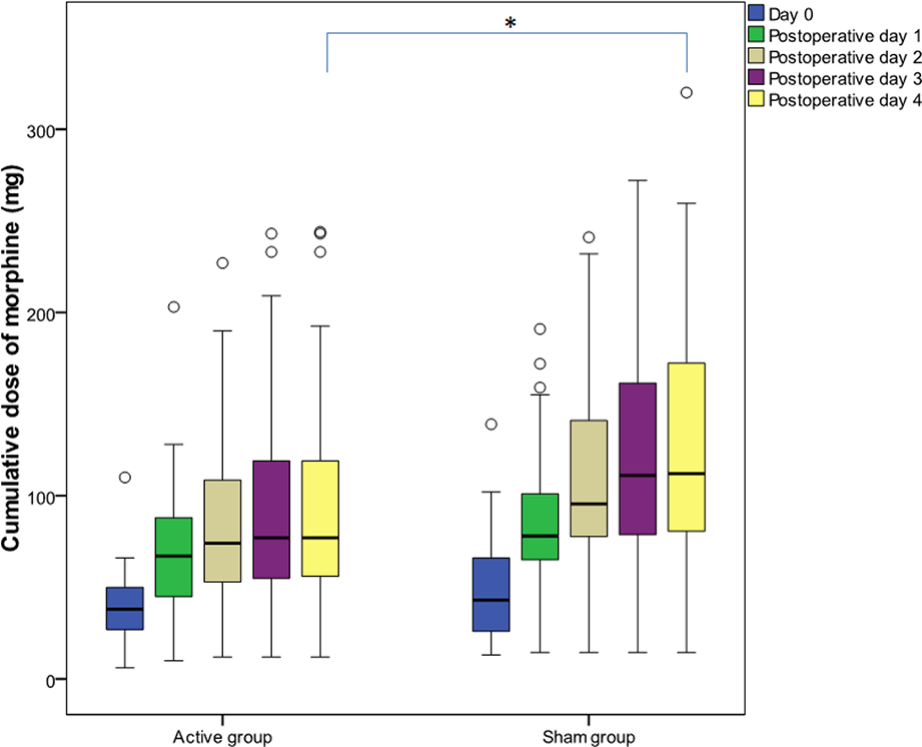
Cumulative morphine dose administered during the first 120 h after surgery. Data are presented as median [(interquartile range (IQR)]. (○) represents outliers. Day 0 covers the period after the first transcranial direct current stimulation (tDCS) immediately after surgery until the second tDCS session; postoperative day 1 covers the period after the second tDCS session on the first postoperative day; postoperative day 2 covers the period after the third tDCS session; postoperative day 3 covers the period after the fourth tDCS session; postoperative day 4 covers the period after the fifth tDCS session. After the fifth interventional session, cumulative morphine dose was significantly lower (p = 0.043) in the tDCS group. *p < 0.05.

Cumulative morphine dose administered during the first 120 h after surgery was lower by 31.25% (Cohen’s d = 0.42) in the tDCS group [77.00 (54.00–123.00) vs. 112.00 (79.97–173.35) mg, p = 0.043], and the difference was statistically significant (**Figure 3**). The number of analgesia requests delivered by PCA pump did not differ significantly between groups. Additional analysis comparing cumulative morphine use in men and women in both the tDCS and the sham groups did not show any significant differences between tDCS vs. sham in men or in women. However, cumulative morphine use was significantly higher in men, compared to women, in both the tDCS and the sham group at all time points (**Table 3**).

**Table 3:** Cumulative morphine dose administered in the first 120 h after surgery.

	Active group (n = 27)		Sham group (n = 28)		Female (tDCS vs. sham), p value	Male (tDCS vs. sham), p value	Active group (female vs. male), p value	Sham group (female vs. male), p value
	**Female (n = 11)**	**Male (n = 16)**	**Female (n = 5)**	**Male (n = 23)**				
**Day 0**	30 (8–44)	43 (31.75–54.5)	20 (16–47)	44 (35–68)	0.913	0.454	0.050*	0.039*
**Day 1**	50 (28–68)	86.5 (64.25–105.8)	46 (36.5–73.5)	80 (68–121.5)	0.827	0.601	0.007*	0.019*
**Day 2**	68 (29–79.3)	98.5 (66.25–138.88)	57 (45–79)	103 (88.45–150)	1.000	0.373	0.008*	0.004*
**Day 3**	68 (29–79.3)	106.5 (66.5–168.90)	71.3 (48–87.55)	115 (89–174)	0.661	0.404	0.008*	0.007*
**Day 4**	68 (29–79.3)	106.5 (66.5–186.80)	76.3 (53–95.55)	123.8 (89–174.95)	0.441	0.373	0.008*	0.016*

Data are presented as median [interquartile range (IQR)]. Day 0 covers the period after the first transcranial direct current stimulation (tDCS) immediately after surgery until the second tDCS session; postoperative day 1 covers the period after the second tDCS session on the first postoperative day; postoperative day 2 covers the period after the third tDCS session; postoperative day 3 covers the period after the fourth tDCS session; postoperative day 4 covers the period after the fifth tDCS session. Compared to men, cumulative morphine dose was significantly lower in women in both the tDCS and in the sham group at all times.

*p < 0.05 (Mann–Whitney U test).

**Figure 4:**
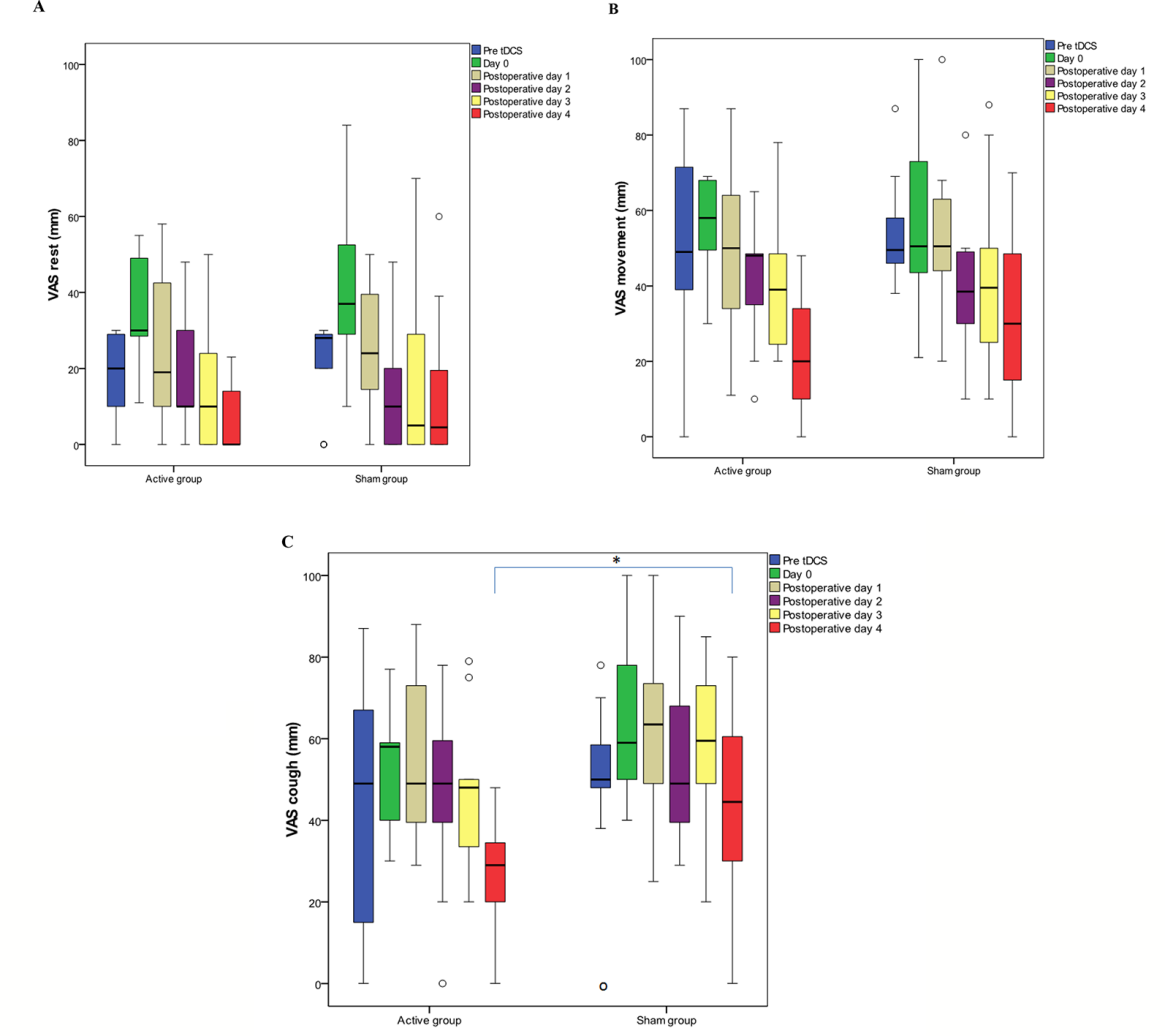
Maximum visual analog scale (VAS) pain scores during rest **(A)**, movement **(B)**, and cough **(C)**. Data are presented as median (IQR). (○) represents outliers. Day 0 covers the period after the first transcranial direct current stimulation (tDCS) immediately after the surgery until second tDCS session; postoperative day 1 covers the period after the second tDCS session on the first postoperative day; postoperative day 2 covers the period after the third tDCS session; postoperative day 3 covers the period after the fourth tDCS session; postoperative day 4 covers the period after the fifth tDCS session. **(C)** Significantly lower (p = 0.018) maximum VAS pain score with cough was recorded after the fifth interventional session in tDCS group. * p < 0.05.

On postoperative day 5, maximum VAS pain score with cough was lower by 34.83% in the tDCS group [29.00 (20.00–39.00)] vs. sham stimulation [44.50 (30.00–61.75)], p = 0.018; this difference was confirmed by PROs, where interference of pain with cough was 80.00% lower [10.00 (0.00-30.00) vs. 50.00 (0.00-70.00), p=0.013] (**Figure 4C**). There was no significant difference in maximum VAS pain scores during rest and movement, between the tDCS and the sham group (**Figures 4A**, **B**). Also, no difference in maximum VAS pain scores was found in men compared to women in both the tDCS and the sham group.

We did not record any complications from tDCS, except for “itching” and “tingling” in the control group. Postoperative complications included respiratory complications, arrhythmias, surgical complications, and infections but there was no significant difference between groups (p = 0.522).

There was no significant difference between groups regarding anxiety, mood, and presence of depression (**Table 4**). While interference of pain with cough was significantly lower in the active tDCS group, we did not identify any other differences existed between groups with regard to PRO (**Table 5**). Data at discharge were not significantly different between groups (**Table 6**).

**Table 4:** Anxiety level, mood and Beck depression scores preoperatively and on the day of discharge.

Variable	Active group (n = 27)	Sham group (n = 28)	p Value
**Anxiety VAS-A (mm)**			
**Preoperatively**	20.00 (00.00–41.00)	19.50 (00.00–30.00)	0.793^c^
**Discharge**	0.00 (00.00–00.00)	0.00 (00.00–00.00)	0.634^c^
**Mood VAS (mm)**			
**Preoperatively**	85.00 (60.00–95.00)	89.50 (82.25–97.25)	0.294^c^
**Discharge**	100.00 (95.00–100.00)	100.00 (90.00–100.00)	0.507^c^
**Beck depression scale (points)**			
**Preoperatively**	4.00 (1.00–6.00)	2.00 (0.00–5.00)	0.396^c^
**Discharge**	5.00 (1.00–7.00)	3.00 (0.50–7.00)	0.465^c^

Data are presented as median (IQR). Data analysis did not show any significant differences between the active vs. sham group.

c Mann–Whitney test.

**Table 5:** Patient-reported outcomes on postoperative day 1 and day 5.

Variable	Active group (n = 27)	Sham group (n = 28)	p Value
**Maximal VAS-P (mm)**			
**Day 1**	40.00 (30.00–70.00)	55.00 (40.00–70.00)	0.339^c^
**Day 5**	50.00 (27.50–70.00)	50.00 (40.00–80.00)	0.473^c^
**Percentage of time in severe pain (%)**			
**Day 1**	5.00 (5.00–10.00)	10.00 (5.00–10.00)	0.220^c^
**Day 5**	20.00 (10.00–30.00)	30.00 (10.00–45.00)	0.342^c^
**Pain interference with movement**			
**Day 1**	10.00 (0.00–30.00)	7.50 (0.00–17.50)	0.409^c^
**Day 5**	20.00 (0.00–50.00)	40.00 (0.00–60.00)	0.394^c^
**Pain interference with cough**			
**Day 1**	0.00 (0.00–5.00)	5.00 (0.00–10.00)	0.160^c^
**Day 5**	10.00 (0.00–30.00)	50.00 (0.00–70.00)	0.013^c^
**Pain interference with sleep**			
**Day 1**	0.00 (0.00–0.00)	0.00 (0.00–0.00)	0.806^c^
**Day 5**	10.00 (00.00–30.00)	20.00 (0.00–60.00)	0.334^c^
**Satisfaction with pain management**			
**Day 1**	100.00 (80.00–100.00)	100.00 (80.00–100.00)	0.919^c^
**Day 5**	100.00 (90.00–100.00)	100.00 (80.00–100.00)	0.271^c^

Data are presented as median (IQR). Pain interference with cough was significantly higher in the sham group on day 5 (p < 0.013).

c Mann–Whitney test.

**Table 6:** Postoperative data; VAS-pain (P) on discharge, hospital length of stay, and presence of drain.

Parameter	Active group (n = 27)	Sham group (n = 28)	p Value
**Hospital length of stay (days)**	7.00 (6.50–8.00)	8.00 (7.00–11.25)	0.126^c^
**Drain length of presence (days)**	3.45 (2.00–4.50)	2.50 (1.00–4.00)	0.166^c^
**VAS-P rest (mm)**	0.00 (0.00–0.00)	0.00 (0.00–0.00)	0.372^c^
**VAS-P movement (mm)**	10.00 (0.00–25.00)	0.00 (0.00–30.00)	0.653^c^
**VAS-P cough (mm)**	20.00 (0.00–35.00)	8.00 (0.00–30.00)	0.349^c^

Data are presented as median (IQR). Data analysis did not show any significant differences between the active vs. sham group.

c Mann–Whitney test.

From the time tDCs was discontinued until discharge, the number of patients treated with opioids was not significantly different between groups: 4 (14.8%) patients in tDCS group and 7 (25%) in sham group (p = 0.544). Additionally, there was no significant difference in the amount of morphine used in tDCS vs. sham group patients (20.00 (4.25–54.50) mg vs. 8.00 (3.00–30.90) mg, (p = 0.788). The number of patients treated with nonsteroidal anti-inflammatory drugs was 6 (22.2%) in tDCS group vs. 3 (10.7%) in sham group (p = 0.295).

No patient contacted us after discharge due to pain problems. The phone interview conducted 1 year after surgery revealed that 9 of 21 (43%) patients in the tDCS group and 11 of 22 (50%) patients in the sham group had chronic pain (p = 0.87). Timing of chronic pain appearance was not significantly different between groups (p = 0.409): pain was persistent from the discharge in 4 (14.8%) tDCS patients and in 6 (27.3%) sham patients. Pharmacologic chronic pain treatment was needed by 6 (28.6%) patients in tDCS group vs. 8 (36.4%) patients in the sham group (p = 0.444), but only one patient in the tDCS group required opioid therapy. Pain influence on daily activities 1 year after surgery was reported by two patients in the sham group (p = 0.299).

## Discussion

Our results suggest that three to five anodal tDCS sessions on consecutive post-operative days over the left primary motor cortex (Brodmann areas 4) as an adjunct to IV morphine PCA significantly reduce cumulative morphine use, pain with cough and interference of pain on cough in patients with acute pain after thoracotomy.

The use of opioid consumption given by IV PCA as the primary outcome in our study is based on the principle that all patients should have access to good quality postoperative analgesia. Several published studies (Borckardt et al., 2008; Borckardt et al., 2013; Glaser et al., 2016; Khedr et al., 2017b) have used tDCS as component of a multimodal analgesia regimen that also included IV opioids for pain after several different types of surgical procedures and have demonstrated significant reduction of cumulative PCA opioid use, comparable to our findings. A study published by Ribeiro et al. (2017) reported the best result with 73.25% reduction of opioid analgesic use with preoperative application of two tDCS sessions in patients undergoing hallux valgus surgery. However, the Ribeiro study included data collected from patient diaries after hospital discharge (Ribeiro et al., 2017), whereas opioid consumption data in our study as well as in several other hospital-based studies were collected in an objective manner, from PCA pumps in a hospital environment. The opioid sparing effect of M1-tDCS can be explained by acute motor cortex neuromodulation and direct increase of regional endogenous opioid release (Dossantos et al., 2012; Dossantos et al., 2014). Dossantos et al. (2012) have shown that acute tDCS reduces morphine (µ) opioid receptor (MOR) availability in pain-related regions during single-session tDCS in postherpetic neuralgia. The decreased binding of exogenous ligand during PET scan was explained as MOR occupancy by endogenous opioids (Maarrawi et al., 2007). However, Khedr et al. (2017b) suggested that the opioid sparing effect of tDCS could be the result of tDCS boosting the opioid effect or of reduced endogenous perception of pain.

The variable opioid-sparing effect in different tDCS studies can be explained by high heterogeneity of study protocols (twice daily vs. once daily), use of different cortical targets (motor cortex vs. prefrontal cortex), different non-opioid analgesics, including metamizole, paracetamol or ketorolac, different types of surgical procedures, and localization in the human body. Some studies used a combination of spinally delivered local anesthetic and opioid for intraoperative analgesia and postoperative systematic opioid (Khedr et al., 2017b; Ribeiro et al., 2017), while other studies, including our study, used perineural local anesthetic administration as adjunct to systemic opioid analgesia (Borckardt et al., 2013). Perineural local anesthetics (femoral nerve block, intercostals block) and systemic non-opioid analgesics (nonsteroidal and steroidal anti-inflammatory drugs) act primarily by peripheral mechanisms (Eisenach and Brennan, 2018), while spinally administered drugs and systemic opioids act primarily in the central nervous system (Eisenach and Brennan, 2018). Based on the works of Nitsche et al. (2003a) and Alonzo et al. (2012), different medications can influence tDCS efficacy, thereby influencing the results of clinical trials. The influence of multiple perioperative analgesics and regional analgesia techniques on tDCS efficacy needs to be explored in further studies.

In our study we tried to overcome some of these issues by excluding patients with chronic pain and preoperative opioid use. In an attempt to use fewer medications, propofol, and remifentanil were used for anesthesia, combined with a single intercostal block and a single dose of paracetamol intraoperatively, followed by postoperative analgesia using morphine IV-PCA. During the intraoperative period there was no difference in the amount of remifentanil infused for intraoperative analgesia and in the amount of morphine used to provide analgesia at rest (VAS < 30 mm), before the first intervention session in the immediate postoperative period. In the postoperative period, paracetamol was given only when a patient requested additional analgesia, and none of the patients requested it. However, despite well-defined exclusion criteria, our patients were still using different medications for treatment of chronic comorbidities.

Propofol was chosen as primary anesthetic because data suggest it confers neuroprotective effect against intercostal nerve injury after thoracotomy (Song et al., 2012) and may also have a role in preventing remifentanil-induced hyperalgesia (Song et al., 2012). Remifentanil was chosen for intraoperative use in order to minimize the impact of intraoperative opioid administration on postoperative analgesia and on the need for postoperative opioids. Morphine administration started at the time of the “air leak test” (testing of the bronchial stump and lobe for air leak), usually 40 min before closing the chest (Munoz et al., 2002).

Cumulative opioid use in our study showed divergent trajectories between the two groups, with the divergence getting wider after the second tDCS session, a finding similar to the results reported by Khedr et al. (2017b). Other studies have shown that the effect of tDCS becomes obvious 12 h after the first tDCS session or after two tDCS sessions (Glaser et al., 2016; Borckardt et al., 2017). Borckardt et al. (2017) have suggested that tDCS should be used immediately after surgery in order to maximize its effect, but in our study and in several other studies the first tDCS session started 30 min to 3 h after wound closure. In contrast to earlier studies which did not precisely evaluate pre tDCS pain intensity, the first tDCS session was started in our study only after patients received postoperative analgesics and VAS pain at rest was ≤30 mm, in order to have comfortable patients with similar pain intensity at the time of tDCS. The number of analgesia requests was recorded; however, PCA settings were adjusted in response to patients’ needs by increasing the bolus dose of morphine from 1 to 2 mg, and this is why there was no difference in the number of analgesia requests. Therefore, the amount of morphine used (not the number of analgesia requests) better reflects the quality of analgesia in this patient population.

While the Ribeiro et al. study only included female patients, our study included both men and women. When analyzing data in male patients only and in female patients only, we did not find any significant difference between tDCS vs. sham groups with regard to cumulative morphine use and maximum pain scores. However, when comparing male vs. female patients, data analysis showed significantly higher cumulative morphine use in male patients at all time points in the postoperative period. Published studies have not shown sex to be a consistent predictor of analgesic efficacy of morphine: some studies found reduced morphine efficacy (Cepeda and Carr, 2003; Miller and Ernst, 2004) or increased morphine efficacy (Niesters et al., 2010) in female patients, while other studies showed no difference (Fillingim et al., 2005), and there is a hypothesis that different genotypes, especially polymorphism of OPRM1 gene responsible for MOR synthesis (Janicki et al., 2006; Sia et al., 2008) might play an important role.

In our study, VAS pain scores at rest, with movement and with cough were not different between the two groups, but cumulative opioid use was significantly lower in the tDCS group. This finding is in agreement with the findings reported by Khedr et al. (2017b) and suggests that tDCS has a significant analgesic and opioid-sparing effect. However, because the main outcome of our study was cumulative opioid use, the study was not designed and did not have adequate power to assess differences in postoperative VAS scores between patient groups. Therefore, we decided to analyze the maximum value of pain during cough, i.e., “the worst possible pain” which, as suggested by Jensen et al. (2015) is more important than “average pain” for valid assessment of the treatment effect. In the tDCS treated group, maximum VAS pain during cough was significantly lower after the fifth tDCS session on postoperative day 5. This finding can be explained by a cumulative effect of repeated tDCS, as reported in tDCS studies on healthy volunteers and patients with chronic pain (Lima and Fregni, 2008; Alonzo et al., 2012).

Our decision to include in data analysis only patients who had three or more tDCS sessions was based on the concept of cumulative tDCS effect. Driven by the positive benefits of repeated rTMS sessions for the treatment of depression, many trials have applied the same concept using tDCS. Clinical trials have applied repeated tDCS sessions with the assumption that repeated stimulation will result in cumulative and sustained changes in cerebral function (Mori et al., 2010). Recent evidence suggests that the excitatory effects derived from tDCS might be cumulative both at the cortical (Alonzo et al., 2012) and at the behavioral level in healthy people as well as in different patient populations (Boggio et al., 2008a; Alonzo et al., 2012). Furthermore, tDCS alters cortical excitability more effectively when given daily rather than every other day over a 5-day period (Alonzo et al., 2012). Most studies showing an effect of tDCS on opioid consumption have design similar to our study, using four tDCS sessions, either as two sessions per day for 2 days or as one session per day for four consecutive days (Borckardt et al., 2013; Glaser et al., 2016; Khedr et al., 2017b), whereas two studies used only one tDCS session with different results (Borckardt et al., 2011; Dubois et al., 2013). Based on this knowledge, data analysis in our study was performed “per protocol”.

We believe that the 34.83% reduction of maximum VAS pain during cough observed after the fifth tDCS session is clinically significant, based on mean value which stratifies tDCS group patients with mild pain compared to moderate pain in sham group (Jensen et al., 2003; Younger et al., 2009).

Some published studies have not shown a benefit from tDCS with regard to pain scores (Khedr et al., 2017b). The best results have been reported with preoperative tDCS application (Ribeiro et al., 2017), but in that study data were recorded and collected by patients at home. The model of post-thoracotomy pain used in our study is challenging due to the complex origin of pain, including nociceptive (skin incision), somatic (muscles, ribs) caused by retraction and deeper tissue trauma, resection of ribs and dislocation of costovertebral joints, visceral (pleura) caused by chest tube irritation, and nerve involvement due to intercostal nerve injury and inflammation (Ochroch and Gottschalk, 2005). Intercostal nerves, and the vagus and phrenic nerves are all responsible for nociceptive input after thoracotomy. Intercostal nerve injury due to incision, retraction, or suture can have significant impact on post-thoracotomy pain (Benedetti et al., 1998), whereas phrenic nerve activity can cause shoulder pain (Scawn et al., 2001). Further work is needed to evaluate whether other surgical procedures are “responding” to the analgesic effect of tDCS.

Postoperative pain is complex and not a single entity. Use of tDCS in patients with chronic pain state (fibromyalgia) can result in mood improvement (Khedr et al., 2017a), whereas studies investigating acute postoperative pain, including our study, did not find a difference in mood and depression scores between patients treated with tDCS vs. sham (Borckardt et al., 2013; Khedr et al., 2017a). The different effect of tDCS on mood and depression scores in patients with acute vs. chronic pain can be explained by the different pain model, stimulation electrode location, lower number of tDCS sessions, and, perhaps more important, other psychological factors, including catastrophization, which are not evaluated in clinical acute pain studies (Borckardt et al., 2013; Khedr et al., 2017a; Lefaucheur et al., 2017).

In our study, tDCS showed short-term benefit as evidenced by significant reduction of morphine use. However, patient follow-up during hospital stay and the follow-up phone interview 1 year later did not show a difference between groups with regard to analgesic medication use and incidence of chronic pain, which were not the primary aims of the study. This short-term beneficial effect of tDCS could still be clinically relevant as part of multimodal post-thoracotomy pain treatment in cases where regional techniques have failed or are contraindicated. Because the severity of acute post-thoracotomy pain has been associated with chronic pain after surgery (Bayman et al., 2017; Niraj et al., 2017), different analgesic techniques, especially non-pharmacological opioid-sparing techniques with minimal side effects should be explored for their utility in clinical practice.

Strengths of our study include the use of a nonpharmacological and safe technique as part of multimodal analgesia regimen for a very painful surgery (thoracotomy), prospective study design, small but adequate sample size, sound blinding and randomization procedures, and a clinically meaningful primary outcome. The finding of reduced “worst possible pain” during cough is clinically important for patients in the postoperative period and is especially useful after video assisted thoracoscopic surgery (VATS) and in cases where epidural analgesia is contraindicated.

Limitations of our study include the small (but adequate, based on sample size calculation) number of patients, and the absence of preoperative qualitative sensory testing which could provide additional insight into the character of patient distress. The fifth intervention session was performed in 11 patients in the tDCS group and 16 patients in the sham group, therefore we calculated Cohen’s criteria of d 0.42, which shows that sample size has low impact on results.

Although previous publications have used two tDCS sessions per day (Borckardt et al., 2013; Glaser et al., 2016; Borckardt et al., 2017), we chose to use single tDCS session repeated daily based on previously published data (Borckardt et al., 2011; Khedr et al., 2017a). From our point of view, daily application of tDCS stimulation seems more feasible in the real world of a busy hospital.

The short-term (5 days) follow-up period is a significant limitation of this study, but was based on average hospital stay after thoracotomy in our Institution. This was a “proof of concept” study, as there are no published data about the effects of tDCS as part of multimodal management of acute post-thoracotomy pain. However, after patients leave the hospital, our experience showed that it is difficult to achieve high patient response rates to phone interviews and patients are generally not willing to participate in repeated follow-up audits. Therefore, in order to respect the wishes of our patients to only be contacted once after surgery, we determined that a 1-year follow-up period was the most suitable.

Recently, tDCS has been increasingly studied as an effective pain treatment technique (Woods et al., 2016). This type of non-invasive modulation has been studied previously in multiple target brain areas including the primary motor cortex, dorsolateral prefrontal cortex, and less frequently cerebellum (O'connell et al., 2014). In our study we decided to stimulate the primary motor cortex, since this pattern of stimulation has proven to be successful in a number of previous studies. This choice of target region is based on presumed modulation of the lateral thalamus hyperactivity by input from cortico-thalamic projections originating from the primary motor cortex, which is considered to be related to sensory-discriminative information processing (Boggio et al., 2008b). Considering limitations related to tDCS methodological issues, it should be noted that in this study we chose a pattern of stimulation based on the experience of most previous studies, applying standard electrode positioning (C3-Fp2), which, may have contributed to reduced stimulation efficacy. On the contrary, we opted for a relatively strong and focal stimulation, especially considering that these patients receive analgesics as primary therapy after surgery, and tDCS can be tolerated without significant side effects. It is also worth noting that with tDCS montage as applied, the return electrode positioned over the right prefrontal cortex (Fp2) could also contribute to pain alleviation. Moreover, studies on the processing of painful information in the brain, in addition to activation of the thalamus, insular cortex, anterior cingulate cortex, and primary and secondary areas of the somatosensory cortex, also indicate activation of the prefrontal cortex (Ong et al., 2019). Therefore, in future research, the protocol could be systematic shaping, with possible consideration of another anode position (C1 position would probably be the most suitable area for the thoracic area, instead of C3 as we used), and the choice of hemisphere stimulation could be adjusted so that tDCS can always be applied on the contralateral side, depending on the side of the chest where thoracotomy was performed.

Nonpharmacological techniques can be viewed as measures aiming to reduce postoperative opioid use. Resources among hospitals vary widely and not all analgesia modalities are available in every hospital; therefore, exploration of the appropriate use of certain analgesic techniques can widen the panel of analgesia techniques available and result in improved hospital analgesic protocols. We suggest that future studies could define proper timing for a preoperative tDCS session because there are data suggesting that preoperative tDCS can also be beneficial (Ribeiro et al., 2017).

In conclusion, our study suggests that postoperative daily tDCS confers significant benefits with regard to postoperative analgesia after thoracotomy, and this benefit is probably applicable not only for open thoracic surgery, but also for postoperative pain management after VATS, when planned thoracoscopic procedures require conversion to open surgery and after emergency thoracotomy. Because the conclusion about lack of long-term tDCS efficacy on pain in our study is based only on one telephone survey conducted 1 year after surgery, we suggest that future studies could better explore possible tDCS effects on chronic postoperative pain presence by also collecting data about pain 3 and 6 months after surgery.

## Data Availability Statement

The datasets generated for this study are available on request to the corresponding author.

## Ethics Statement

The studies involving human participants were reviewed and approved by the Institution Ethics Committee of Military Medical Academy. The patients/participants provided their written informed consent to participate in this study.

## Author Contributions

DS initiated study design, collected data, interpreted data, did literature search, and wrote the manuscript. KM collected data, interpreted data, and wrote the manuscript. NR performed statistical data analysis, did literature search, and collected data. VC did literature search, collected data, and performed the surgery. NM did literature search, collected data, and performed the surgery. VN did literature search, collected data, and performed the anesthesia. SZ did literature search, collected data, and performed the anesthesia. MK interpreted data, did literature search, and revised the manuscript. TI initiated study design, interpreted data, and revised the manuscript. All authors read and approved the final manuscript.

## Funding

This work was supported by a grant from the Ministry of Defense of the Republic of Serbia (Project MFVMA/05/19-21) for TVI.

## Conflict of Interest

The authors declare that the research was conducted in the absence of any commercial or financial relationships that could be construed as a potential conflict of interest.
